# Physical workload, long-term sickness absence, and the role of social capital. Multi-level analysis of a large occupation cohort

**DOI:** 10.5271/sjweh.3874

**Published:** 2020-07-01

**Authors:** Eszter Török, Alice Jessie Clark, Annette Kjær Ersbøll, Jakob Bue Bjorner, Andreas Holtermann, Reiner Rugulies, Anthony D LaMontagne, Allison Milner, Naja Hulvej Rod

**Affiliations:** Department of Public Health, University of Copenhagen, Denmark; Copenhagen Stress Research Center, Copenhagen, Denmark; National Institute of Public Health, University of Southern Denmark, Copenhagen, Denmark; National Research Centre for the Working Environment, Copenhagen, Denmark; Department of Sports Science and Clinical Biomechanics, University of Southern Denmark, Odense, Denmark; Department of Psychology, University of Copenhagen, Copenhagen, Denmark; Determinants of Health Domain, Institute for Health Transformation, School of Health & Social Development, Deakin University, Geelong, Victoria,Australia; Centre for Health Equity, Melbourne School of Population and Global Health, University of Melbourne, Melbourne

**Keywords:** Key terms buffer, collaboration, effect modification, healthcare, justice, stress, trust, work environment

## Abstract

**Objectives:**

This study determined the prospective relation between physical workload and long-term sickness absence (LTSA) and examined if work-unit social capital may buffer the effect of high physical workload on LTSA.

**Methods:**

We included 28 925 participants from the Danish Well-being in HospitAL Employees (WHALE) cohort, and followed them for two years. Physical workload and social capital were self-reported and categorized into low, medium, and high. Physical workload was analyzed on the individual level, whereas social capital was analyzed on the work-unit level. LTSA data were obtained from the employers’ payroll system. We performed two-level logistic regression analyses: joint-effect and stratified analyses adjusted for baseline covariates.

**Results:**

High versus low physical workload was associated with a higher risk of LTSA [odds ratio (OR) 1.55, 95% confidence interval (CI) 1.40–1.72]. There was a multiplicative interaction (P=0.007) and a tendency of sub-additive interaction [relative excess risk due to interaction (RERI) -0.49, 95% CI -1.03–0.06] between physical workload and social capital. Doubly exposed employees had the highest risk of LTSA (OR 2.45; 95% CI 2.02–2.98), but this effect was smaller than expected from the sum of their main effects.

**Conclusions:**

We found a prospective relation between physical workload and LTSA but no evidence of high social capital buffering the effect of high physical workload. High physical workload was a risk factor for LTSA at all levels of social capital and employees exposed to both exposures had the highest risk of LTSA. Interventions should aim at both improving social capital and reducing physical workload in order to efficiently prevent LTSA.

There is evidence that physically strenuous work such as extreme bending or twisting of the neck and back, monotonous movements, working standing and squatting, pushing, pulling, and lifting heavy loads is associated with higher rates of sickness absence (1–8). A range of psychosocial work factors such as role conflicts, high emotional demands, low job control, and low supportive leadership have been also found to increase sickness absence risk (4, 9–11).

In contrast, high level of social capital (SC) at work has been shown to be beneficial for employee health (12, 13). SC has various definitions but has generally been described as a resource that is accessible for groups and individuals via their network (14, 15). Despite a universal definition, the main components of SC are social networks, trust, and civic norms (16, 17). SC has been defined and measured both as an individual and group characteristic, yet the most common approach is to treat it as a collective resource (18). In a workplace setting – with various social interactions and networks – SC can be considered an important element of the psychosocial working environment, comprising elements of trust, justice, and collaboration (16).

Workplace SC is particularly relevant in the healthcare sector where it is vital that employees foster trust and cooperative efforts to achieve common goals (19). In a previous study among healthcare employees, we demonstrated that high work-unit SC is associated with a lower risk of long-term sickness absence (LTSA) (20).

Given that physical and psychosocial work factors intersect, studies have shifted towards examining the effect of potential interactions between these exposures on various health outcomes (21). Studies have found that the exposure to physical work demands combined with negative psychosocial exposures further increased the risk of adverse health effects (5, 22, 23). Others investigated the role of social relations in physically demanding work and reported that support from supervisors and co-workers can buffer the negative health effects of physical work demands (24, 25). In such cases, supervisors and co-workers might engage in work strategies that ensure employees are not overburdened.

In the working population, LTSA could be used as a proxy for a range of health (ie, physical and mental) conditions (26). The mechanism through which SC could influence LTSA is yet to be understood, but one hypothesis is that high SC modifies the effect of adverse exposures such as high physical workload (PW). For example, SC could play a buffering role through psychosocial processes: via trusting and respectful relations, employees could better access affective, instrumental, and informational support from their co-workers and/or supervisors, which could enhance their control and reduce stress (27). Furthermore, SC could also reduce the physiological stress response to high PW via lower cardiovascular reactivity (ie, blood pressure and heart rate), better endocrine (lower cortisol and higher oxytocin response) and immune function (28).

In the present study, we aimed to determine the prospective relation between PW and LTSA risk and study whether high SC may buffer the effect of high PW on LTSA. We hypothesized that the risk of LTSA associated with high PW is lower in work-units with high SC than in those with low SC. Further, we hypothesized that the combined exposure group – with high PW and low SC – may be at highest risk of LTSA.

## Methods

### Study population

The study is based on the Well-being in HospitAL Employees (WHALE) study, a prospective observational cohort study including data on the physical and psychosocial work environment of healthcare employees in the capital region of Denmark (29). In 2014, 37 720 employees were invited to participate in the self-administered survey. The response rate was 84% (ie, 31 823 employees).

Participants were excluded due to missing personal identification number (N=151); duplicate responses (N=82); missing information on covariates (N=555); responding “I don’t know/not relevant” to the information on PW (N=1397); being employed in work-units with <3 employees (N=524); and not being actively employed at baseline (N=189). In total, this yielded a final study population of 28 925 participants nested within 2267 work-units who were followed for two years from baseline (March 2014).

### Physical workload

PW was measured by the following three items: (i) “Are you affected by heavy lifting and relocations in your daily work?” (ii) “Does your work require pulling and pushing (for example beds, carts or anything else) during your daily work)?” and (iii) “Does your work require monotonous postures and movements during your daily work?” The response options were: “to a very high degree”, “greatly”, “somewhat”, “to a small extent”, “not at all”. Of the mean score of the three items, a summary scale of PW was composed (Cronbach’s alpha=0.74). The scale ranged from 0–100, with higher scores representing higher PW. Finally, PW was categorized into “low”, “moderate”, and “high” based on the tertile cut-off points.

### Social capital

In line with previous Danish studies (29, 30), SC was measured using a global measure covering trust, justice and collaboration (ie, indicators of SC) among employees and between employees and their supervisors (13, 31, 32). The measure was constructed of the following eight items: (i) “Do you get help and support from your colleagues when needed?”, (ii) “Does the management trust the employees to do their work well?”, (iii) “Do you trust the information that comes from the management?”, (iv) “Is the work distributed fairly?”, (v) “Are conflicts resolved in a fair way?”, (vi) “Do you and your colleagues take responsibility for a nice atmosphere and tone of communication?”, (7) “Are you and your colleagues good at coming up with suggestions for improving work procedures?” (viii) “Is your staff group respected by the other staff groups at the workplace?”. The response scale was a 5- or 7-point Likert scale ranging from “not at all” to “to a great extent”.

A scale ranging from 0–100 was computed from the mean of the item responses (Cronbach’s alpha=0.85). SC was recorded as missing if >50% of the item responses were missing. In all work-units of at least three employees, work-unit SC was calculated as the mean SC score of individuals belonging to the same work-unit, and it was recorded as missing if <50% of eligible employees in a given unit had individual data on SC. Based on the quartile cut-offs, work-unit SC was categorized into (i) low, (ii) medium, and (ii) high (25%, 25–75%, 75%, respectively).

### Covariates

Covariates were identified according to prior knowledge and the methods of directed acyclic graphs (33). Sex, age, seniority, occupational group (doctors/dentists, nurses/nurse assistants, other health staff, educational staff, service and technical staff, and administrative staff), and working hours (full time versus part-time) were added as individual level covariates, and work-unit size (ie, number of actively employed within the units at baseline), the proportion of female employees, and the proportion of part-time work as work unit-level covariates. The proportion of female employees and proportion of part-time work are characteristics that might be relevant in terms of the level of work-unit SC. For example, women may rate measures of trust and collaboration differently than men (a similar argument could apply for part- versus full-time employees), and the aggregated SC may therefore be dependent on the proportion of female and part-time workers in the work-units. Information on all covariates were drawn from the payroll system, except from work-unit size, which was obtained from the baseline survey.

### Long-term sickness absence

This study specifically focuses on LTSA, reflecting the long-term health consequences (both physical and mental conditions) of PW. We linked the survey responses in 2014 to sickness absence data from the employers’ payroll system. LTSA was operationalized as an episode of ≥29 consecutive days of sickness absence within two years following baseline, and it was used as a binary outcome variable.

### Statistical analyses

In order to estimate how much of the variation in individual SC was explained by the work-units, the intraclass correlation coefficient (ICC) was computed by adding individual SC as the outcome variable and work-unit as a random effect into the regression model. The ICC was 24%, much higher than in another recent Danish study (34), indicating that a considerable amount of variation in SC is accounted for by the work-units. This level of clustering gives a justification for the use of aggregated SC in the analyses. The prospective relation between PW and LTSA risk was estimated in two-level logistic regression analyses with work-unit as a random effect (ie, random intercept model). First, we fitted an age- and sex-adjusted model, then a model with further adjustment for SC, followed by a multiple adjusted model including PW, SC, and all baseline covariates. SC was conceptualized as a group-level property instead of being an individual characteristic. Thus, measuring and analyzing SC on the work-unit level (while taking into account that employees were nested within work-units) seemed to be the most appropriate choice and therefore, individual SC was not accounted for in the analyses. Second, we decomposed the PW scale into its individual components (ie, heavy lifting/relocation, pulling/pushing, and monotonous postures) and their individual main effects on LTSA were also analyzed.

Given that the model we used (ie, logistic regression) is a multiplicative model, the potential interaction effect was first tested by adding a product term of PW and SC into the regression model. This yielded a P-value for multiplicative interaction. Furthermore, in line with the STROBE recommendations, we performed joint effect analyses (35, 36) to determine the combined effect of high PW and low SC on LTSA risk, with those exposed to low PW and high SC as the joint reference category. As additive interaction is considered to be a relevant public health measure that helps identifying sub-groups of intervention (37), we also evaluated additive interaction by calculating the relative excess risk due to interaction (RERI) in the doubly exposed (high PW–low SC) group. In case of a positive additive interaction (RERI>0), the combined effect of the two exposures exceeds the sum of their individual effects (38). If the interaction is negative (RERI<0), the joint effect is smaller than the individual effects combined. Finally, in addition to the analyses with one reference group, we also estimated the effect of PW (low, moderate, high) within strata of SC (low, medium, high). All analyses were performed with SAS 9.4 (SAS Institute, Cary, NC, USA) using PROC GLIMMIX procedure. The results are presented as odds ratios (OR) and their 95% confidence intervals (95% CI).

In order to test the robustness of the results, we conducted three sensitivity analyses. First, because prior LTSA may affect the level of later PW and is a known risk factor for subsequent sickness absence (39), we excluded all individuals with registered LTSA one year prior to baseline from the analyses. Importantly, prior LTSA could also be a result of high PW experienced previously, meaning that it can be on the causal pathway, thus controlling for prior LTSA may introduce over-adjustment. Therefore, exclusion of those with prior LTSA was only performed in a sensitivity analysis and not in the main analysis. Second, assuming that the vulnerability to PW may differ between men and women, we conducted a sex-stratified analysis. Lastly, in case of small work-units (eg, 4 employees), the aggregated SC was based on only a few individual scores (especially if some were missing), so the reliability of these average scores might be low. Therefore, the investigated association was further tested on a subsample consisting of only work-units with ≥10 employees. In all sensitivity analyses, multiple adjusted models were fitted.

## Results

### Baseline characteristics

Table 1 shows the characteristics of the study population across PW levels. Age at baseline ranged from 18–75 years with a mean of 45 years. The mean age was slightly lower in the high than in the low and moderate PW groups. The majority (79%) were female, with a somewhat higher proportion at the two higher levels of PW. The largest occupational group consisted of nurses/nurse assistants (43%), followed by administrative staff (18%), other health staff (14%) and doctors/dentists (12%). There was a higher proportion of doctors/dentists (21%) in the low PW group and a higher proportion of nurses/nurse assistants (61%) in the high PW group. Regarding working hours, a higher proportion of employees were hired part-time in the high PW (43%) than in the moderate (37%) and low PW (26%) groups.

**Table 1:** Baseline characteristics of 28 925 individuals across levels of physical workload (PW), and baseline characteristics of 2267 work-units across levels of the average physical workload of the work-units. [SD=standard deviation.]

	Total		Physical workload ^a^					
	
	N (%)	Mean (SD)	N (%)	Mean (SD)	N (%)	Mean (SD)	N (%)	Mean (SD)
Individual characteristics ^b^								
Age		45.4 (11.2)		47.5 (10.8)		45.4 (10.8)		43.3 (11.5)
Seniority		10.4 (9.9)		10.5 (9.8)		10.7 (9.9)		10.3 (9.9)
Sex								
Female	22 720 (79)		7859 (75)		6360 (81)		8501 (81)	
Occupational group								
Doctors and dentists	3486 (12)		2236 (21)		972 (12)		278 (3)	
Nurses and nurse assistants	12 321 (43)		2815 (27)		3109 (40)		6397 (61)	
Other health staff	3912 (14)		1538 (15)		1210 (15)		1164 (11)	
Educational staff	890 (3)		402 (4)		238 (3)		250 (2)	
Service and technical staff	3037 (10)		583 (5)		536 (7)		1918 (18)	
Administrative staff	5279 (18)		2973 (28)		1826 (23)		480 (5)	
Working hours								
Part-time ^c^	10 106 (35)		2744 (26)		2883 (37)		4479 (43)	
Work-unit characteristics ^d^								
Social capital		69 (9.0)		71 (8.7)		70 (8.5)		67 (8.9)
Size ^e^		13 (9.9)		9 (6.7)		11 (8.1)		18 (12.1)
Proportion of female	76		71		80		77	
Proportion of part-time work	30		18		32		40	
Proportion of high PW	27		2		14		66	

a PW was categorized into “low”, “moderate” and “high” based on the tertile cut-off points of a scale consisting of 3 items.

b Total (N=28 925); PW low (N=10 457); PW moderate (N=7891); PW high (N=10 487)

c Working <37 hours/week.

d Characteristics across the average PW of the work-units. Total (N=2267); PW low (N=794); PW moderate (N=710); PW high (N=763).

e Actively employed employees at baseline.

In the total population, SC ranged between 26–98 with a mean of 69, and the work-unit size ranged between 3–112 with a mean of 13. SC was slightly lower in the high PW group (67) than in the medium and low PW groups (70 and 71), whereas the average work-unit size was larger in the high PW (18 people) than in the other groups (9, 11).

### PW and the risk of LTSA

During a two-year follow-up, 2856 (10%) employees had ≥1 episode of LTSA. In the multiple adjusted model (table 2), moderate (OR 1.15, 95% CI 1.03–1.28) and high (OR 1.55, 95% CI 1.40–1.72) PW compared to low PW were associated with a higher risk of LTSA.

**Table 2:** Main effect of physical workload (PW) and the individual components of PW on long-term sickness absence (LTSA). [OR=odds ratio; CI=confidence interval; SC=social capital.]

	LTSA ^a^ cases (N=2856)/ subjects (N=28 925)	Sex+age adjusted	Sex+age+SC adjusted	Multiple adjusted ^b^
		
		OR (95% CI)	OR (95% CI)	OR (95% CI)
PW				
Low	866/10 547	1 (Reference)	1 (Reference)	1 (Reference)
Moderate	741/7891	1.17 (1.05–1.30)	1.14 (1.03–1.27)	1.15 (1.03–1.28)
High	1249/10 487	1.59 (1.44–1.75)	1.52 (1.38–1.68)	1.55 (1.40–1.72)
Components of PW ^c^				
Lifting/relocation				
Low	1032/12 156	1 (Reference)	1 (Reference)	1 (Reference)
Moderate	1337/12 715	1.29 (1.18–1.41)	1.27 (1.16–1.39)	1.31 (1.19–1.43)
High	487/4054	1.58 (1.40–1.79)	1.52 (1.34–1.72)	1.56 (1.37–1.77)
Pulling/pushing				
Low	1108/12 943	1 (Reference)	1 (Reference)	1 (Reference)
Moderate	1199/11 379	1.28 (1.17–1.40)	1.26 (1.15–1.38)	1.33 (1.21–1.47)
High	549/4603	1.54 (1.37–1.73)	1.48 (1.32–1.67)	1.55 (1.37–1.76)
Monotonous postures				
Low	560/6331	1 (Reference)	1 (Reference)	1 (Reference)
Moderate	1545/16 101	1.09 (0.98–1.21)	1.07 (0.97–1.19)	1.06 (0.96–1.18)
High	751/6493	1.36 (1.21–1.54)	1.30 (1.15–1.47)	1.27 (1.12–1.43)

a One or more episodes of ≥29 consecutive days of sickness absence.

b Adjusted for age, sex, seniority, occupational group, part-time work, work-unit size, proportion of female employees, proportion on part-time work, PW, and SC.

c The individual PW components are not adjusted for each other in any of the models.

When we analyzed the PW components including lifting/relocation, pulling/pushing and monotonous postures separately, we found for all components that high compared to low exposure was associated with a higher risk of LTSA (table 2).

### Interaction between PW and SC

The results of the analyses with a common reference group, and the effect of PW on LTSA within strata of SC are summarized in table 3. As the results were similar for the sex- and age-adjusted and the multiple adjusted models, only the estimates of the multiple adjusted models were reported. Based on the product term of PW and SC, a statistically significant multiplicative interaction was observed between PW and SC on LTSA risk (P-value for multiplicative interaction=0.007). Using the effect estimates of the doubly and singly exposed groups in the model with a common reference group, the measure of multiplicative interaction was: 2.45/1.91×2.03=0.63, indicating a negative multiplicative interaction.

**Table 3:** Effect of physical workload (PW)and work-unit social capital (SC) on the risk of long-term sickness absence (LTSA) with a common reference category, and the effect of physical workload on LTSA within strata of SC. P=0.0070 (multiplicative interaction). [OR=odds ratio; CI=confidence interval.]

	LTSA ^a^ cases/ number of subjects	Joint-effect with common reference category ^b^	Effect of PW within strata of SC ^c^
	
		OR (95% CI)	OR (95% CI)
High SC			
PW, low	207/3355	1 (Reference)	1 (Reference)
PW, moderate	135/1946	1.15 (0.91–1.44)	1.16 (0.92–1.47)
PW, high	219/1929	2.03 (1.64–2.51)	2.10 (1.66–2.65)
Medium SC			
PW, low	398/4968	1.29 (1.08–1.55)	1 (Reference)
PW, moderate	387/3968	1.62 (1.34–1.95)	1.24 (1.06–1.44)
PW, high	629/5530	2.02 (1.69–2.42)	1.54 (1.33–1.79)
Low SC			
PW, low	261/2224	1.91 (1.56–2.34)	1 (Reference)
PW, moderate	219/1977	1.87 (1.51–2.30)	0.98 (0.81–1.20)
PW, high	401/3028	2.45 (2.02–2.98)	1.30 (1.08–1.57)

a One or more episodes of ≥29 consecutive days of sickness absence.

b Adjusted for age, sex, seniority, occupational group, part-time work, work-unit size, proportion of female employees, proportion on part-time work, PW, and SC.

c Adjusted for age, sex, seniority, occupational group, part-time work, work-unit size, proportion of female employees, proportion on part-time work, and PW.

Furthermore, the estimates showed that employees with high PW and low SC (ie, doubly exposed) had 2.45 times higher odds of LTSA (95% CI 2.02–2.98) than the non-exposed, but the effect size of this joint exposure was lower than expected from the sum of their individual main effects. According to the RERI calculation, there was a tendency of a sub-additive interaction (RERI -0.49, 95% CI -1.03–0.06; P=0.08).

Similarly to the joint-effect model, the results of the stratified analyses showed that those with high PW had a higher risk of future LTSA across all SC strata: the highest risk was estimated in the high SC stratum (OR 2.10, 95% CI 1.66–2.65) followed by the medium (OR 1.54, 95% CI1.33–1.79) and low (OR 1.30, 95% CI 1.08–1.57) SC strata. Nevertheless, given the different baseline risks, the estimates in this model are not directly comparable with each other.

The results of the first two sensitivity analyses are presented in tables 4 and 5. After the exclusion of those with LTSA one year prior to baseline, the effect estimates of both the joint effect analyses and stratified analyses were slightly attenuated but remained in the same direction. Similar to the main analyses (table 3), the highest risk of LTSA was in the high SC stratum (OR 1.99, 95% CI 1.56–2.55), and those exposed to both high PW and low SC had the highest risk of LTSA compared to the non-exposed group (OR 2.38, 95% CI 1.94–2.92), but this effect was smaller than expected based on the effect size of the individual exposures.

**Table 4:** Effect of physical workload (PW) and work-unit social capital (SC) on the risk of long-term sickness absence (LTSA) with a common reference category, and the effect of PW on LTSA within strata of SC among employees without registered LTSA 1 year prior to baseline. P=0.0654 (multiplicative interaction). [OR=odds ratio; CI=confidence interval.]

	LTSA ^a^ cases/ number of subjects	Joint-effect with common reference category ^b^	Effect of PW within strata of SC ^c^
	
		OR (95% CI)	OR (95% CI)
High SC			
PW, low	189/3285	1 (Reference)	1 (Reference)
PW, moderate	122/1897	1.13 (0.89–1.44)	1.14 (0.89–1.46)
PW, high	189/1855	1.92 (1.53–2.41)	1.99 (1.56–2.55)
Medium SC			
PW, low	354/4795	1.28 (1.06–1.55)	1 (Reference)
PW, moderate	330/3794	1.54 (1.27–1.88)	1.19 (1.01–1.40)
PW, high	547/5242	1.97 (1.63–2.38)	1.51 (1.30–1.77)
Low SC			
PW, low	221/2114	1.83 (1.48–2.26)	1 (Reference)
PW, moderate	187/1867	1.80 (1.44–2.24)	0.99 (0.80–1.23)
PW, high	345/2856	2.38 (1.94–2.92)	1.33(1.09–1.63)

a One or more episodes of ≥29 consecutive days of sickness absence.

b Adjusted for age, sex, seniority, occupational group, part-time work, work-unit size, proportion of female employees, proportion on part-time work, PW, and SC.

c Adjusted for age, sex, seniority, occupational group, part-time work, work-unit size, proportion of female employees, proportion on part-time work, and PW.

**Table 5:** Effect of physical workload (PW) on long-term sickness absence (LTSA) within strata of sex. P=0.91 (multiplicative interaction). [OR=odds ratio; CI=confidence interval; SC=social capital.]

	LTSA ^a^ cases/ number of subjects	Effect of PW within strata of sex		

		Age adjusted	Age+SC adjusted	Multiple adjusted ^b^
		
		OR (95% CI)	OR (95% CI)	OR (95% CI)
Male				
PW, low	151/2688	1 (Reference)	1 (Reference)	1 (Reference)
PW, moderate	95/1531	1.21 (0.93–1.59)	1.14 (0.86–1.49)	1.14 (0.86–1.50)
PW, high	183/1986	1.83 (1.44–2.32)	1.63 (1.28–2.08)	1.55 (1.20–2.00)
Female				
PW, low	715/7859	1 (Reference)	1 (Reference)	1 (Reference)
PW, moderate	646/6360	1.16 (1.04–1.30)	1.14 (1.02–1.28)	1.15 (1.02–1.29)
PW, high	1066/8501	1.55 (1.39–1.72)	1.50 (1.35–1.67)	1.54 (1.38–1.73)

a One or more episodes of ≥29 consecutive days of sickness absence .

b Adjusted for age, seniority, occupational group, part-time work, work-unit size, proportion of female employees, proportion on part-time work, PW, and SC.

Furthermore, the results of the analyses stratified by sex revealed no difference between men and women in terms of LTSA risk when exposed to high PW (OR 1.55, 95% CI 1.20–2.00 among men and OR 1.54, 95% CI 1.38–1.73 among women). Lastly, the analyses of including only work-units with ≥10 employees yielded similar results to that of the main analyses. The highest risk of LTSA was in the high SC stratum (OR 2.34, 95% CI 1.74–3.15), and those exposed to both high PW and low SC had the highest risk of LTSA compared to the non-exposed group (OR 2.36, 95% CI 1.86–3.01). These results indicate that excluding smaller work-units does not make a big difference, thus the larger sample size including more individuals working in small work-units was an appropriate choice in the main analyses.

## Discussion

Based on data from a large occupational cohort, we found that high PW is a risk factor for LTSA, but our hypothesis of a protective effect of high work-unit SC among employees with high PW was not supported. There was a statistically significant multiplicative interaction and a tendency for additive interaction between PW and SC, yet the interaction effect was in the opposite direction as theoretically expected. Employees exposed to both high PW and low SC had twice the risk of LTSA than those in positions with low PW and high SC, but the size of this effect was smaller than expected from the sum of their individual main effects.

The result of high PW being a risk factor for LTSA is in line with previous findings that reported a link between physically strenuous work and sickness absence (1–8). There is also some evidence that low SC at work is related to employee ill health. For example, in a previous study in the same population (20), we demonstrated that low SC was a strong risk factor for LTSA. Another previous Danish study reported that among employees with high occupational grade, low workplace SC was associated with higher risk of LTSA (13). Furthermore, studies based on the Finnish Public Sector Study reported that low workplace SC was associated with depression, hypertension, all-cause mortality, and poor self-rated health (12, 27, 40–42).

To the best of our knowledge, this is the first study investigating the potential buffering role of high SC on the effect of high PW on LTSA. The presumed mechanisms linking SC to PW and LTSA are similar to that of social support. Unlike previous studies that found a buffering effect of social support (24, 25), the results of the present study did not confirm such an effect modification by SC in the expected direction. Instead, based on the stratified analyses, the adverse effect of high PW was most pronounced when SC was high. This finding is surprising in light of the existing literature and theoretical considerations, and it needs to be replicated in other studies before drawing any firm conclusions. Furthermore, it is important to note that in the stratified analyses the baseline risk of LTSA is different in the different strata (given the strong main effect of SC) (20), so the effect estimates are not directly comparable across SC strata. In that respect, the results should be interpreted with caution.

A possible explanation for the unexpected findings might be that – in work-units with high SC – there may be a higher social acceptance and support from colleagues and the management for going on sick leave when having symptoms of fatigue or musculoskeletal pain due to high PW. Accordingly, employees in work-units with higher SC are more likely to act (ie, go on LTSA) when needed, compared to relatively less-well-functioning work-units. As fatigue and musculoskeletal disorders are highest in the group with high PW, this could potentially explain the finding of the highest risk for LTSA in the group with high PW and high SC.

Following the results of the analyses with one reference group, among employees experiencing high PW, working in units with high SC is still somewhat better than work-units with low SC. Accordingly, the finding of the sub-additive and multiplicative interaction does not mean that high SC is harmful in any way. Rather, it implies that high SC does not buffer the relation between PW and LTSA, and that the joint effect of the two exposures is less than their main effects combined.

### Strengths and limitations

The major strengths of the study are the high response rate, the large sample size, the register-based follow-up, and the hierarchical structure of the data that enabled the use of an aggregated measure of SC at a relevant work-unit level. A further strength is the prospective study design with a two-year follow-up period, which ensured enough power to adjust for a range of covariates and test the potential effect modification by SC. In addition, we had data available on prior LTSA so we could test the robustness of the findings by including only employees without registered LTSA one year prior to baseline.

A limitation of the study is the lack of objective data (eg, accelerometer data) on PW. The self-reported measurement may have led to some degree of misclassification. For example, factors like generally poorer mental or physical health may have biased the self-reported exposure, resulting in over-reporting of high PW. In a sensitivity analysis, this has been partly addressed by excluding employees who have had at least one episode of LTSA one year prior to baseline. However, this might not have captured more minor or temporary health disturbances of employees, who in turn may have reported the exposure differently. This type of reporting bias (eg, due to poor health conditions) was largely eliminated in case of the exposure to SC as it was aggregated to the work-unit level.

PW was only assessed at one time point in a rather crude manner. For example, it did not contain information about other relevant aspects of the physical work exposure such as the intensity, duration, or frequency of PW. The measure of PW could have been more nuanced and differentiated had these aspects being addressed in the baseline survey. Furthermore, education was not accounted for in the models due to the lack of information on educational level in the data. However, all analyses were adjusted for occupational groups.

Other psychosocial work factors (eg, workplace bullying, workplace violence, job control) were not included as covariates in the analyses, because we suspected that work-unit SC may influence the onset or the level of these factors and therefore, these factors might be mediators in the association between work-unit SC and sickness absence.

An additional limitation is the lack of cause-specific information on LTSA, which would have provided information on whether for example the musculoskeletal and psychological conditions that LTSA is based upon are differently distributed across SC levels. The causes of LTSA would have yielded a more profound knowledge about the underlying mechanism between PW, SC, and LTSA.

### Concluding remarks

We found that high PW is a risk factor for LTSA, but the results did not support a buffering role of high SC. Further, we demonstrated that high PW is a risk factor in all strata of SC with the strongest relative association found in the high SC stratum, although the estimates are not directly comparable given the different baseline risks. Finally, we have also shown that those who are exposed to both high PW and low SC carry more than twice the risk of LTSA than the non-exposed, making them a vulnerable group. Based on our findings, interventions to improve SC in itself are insufficient to effectively prevent LTSA among employees with high PW. Rather, for work groups with high PW and low SC, interventions on both PW and SC are likely to be useful.

### Conflict of interest and funding

The Danish Working Environment Fund supported this work (No. 13-2015-09). The sponsor had no role in the study design, the collection, analysis and interpretation of the data, writing the report or decision to submit it for publication. The authors declare no conflicts of interest.

## References

[1] Hoogendoorn WE, Bongers PM, de Vet HC, Ariëns GA, van Mechelen W, Bouter LM (2002). High physical work load and low job satisfaction increase the risk of sickness absence due to low back pain:results of a prospective cohort study. Occup Environ Med.

[2] Laaksonen M, Pitkäniemi J, Rahkonen O, Lahelma E (2010). Work arrangements, physical working conditions, and psychosocial working conditions as risk factors for sickness absence:bayesian analysis of prospective data. Ann Epidemiol.

[3] Labriola M, Christensen KB, Lund T, Nielsen ML, Diderichsen F (2006). Multilevel analysis of workplace and individual risk factors for long-term sickness absence. J Occup Environ Med.

[4] Labriola M, Lund T, Burr H (2006). Prospective study of physical and psychosocial risk factors for sickness absence. Occup Med (Lond).

[5] Lund T, Labriola M, Christensen KB, Bültmann U, Villadsen E (2006). Physical work environment risk factors for long term sickness absence:prospective findings among a cohort of 5357 employees in Denmark. BMJ.

[6] Sundstrup E, Hansen AM, Mortensen EL, Poulsen OM, Clausen T, Rugulies R (2017). Cumulative occupational mechanical exposures during working life and risk of sickness absence and disability pension:prospective cohort study. Scand J Work Environ Health.

[7] Sundstrup E, Hansen AM, Mortensen EL, Poulsen OM, Clausen T, Rugulies R (2018). Retrospectively assessed physical work environment during working life and risk of sickness absence and labour market exit among older workers. Occup Environ Med.

[8] Voss M, Floderus B, Diderichsen F (2001). Physical, psychosocial, and organisational factors relative to sickness absence:a study based on Sweden Post. Occup Environ Med.

[9] Aagestad C, Johannessen HA, Tynes T, Gravseth HM, Sterud T (2014). Work-related psychosocial risk factors for long-term sick leave:a prospective study of the general working population in Norway. J Occup Environ Med.

[10] Lund T, Labriola M, Christensen KB, Bültmann U, Villadsen E, Burr H (2005). Psychosocial work environment exposures as risk factors for long-term sickness absence among Danish employees:results from DWECS/DREAM. J Occup Environ Med.

[11] Rugulies R, Aust B, Pejtersen JH (2010). Do psychosocial work environment factors measured with scales from the Copenhagen Psychosocial Questionnaire predict register-based sickness absence of 3 weeks or more in Denmark?. Scand J Public Health.

[12] Oksanen T, Kouvonen A, Kivimäki M, Pentti J, Virtanen M, Linna A (2008). Social capital at work as a predictor of employee health:multilevel evidence from work units in Finland. Soc Sci Med.

[13] Rugulies R, Hasle P, Pejtersen JH, Aust B, Bjorner JB (2016). Workplace social capital and risk of long-term sickness absence Are associations modified by occupational grade?. Eur J Public Health.

[14] Bourdieu P, Richardson JG (1986). The Forms of Capital. Handbook of Theory and Research for the Sociology of Education.

[15] Kawachi I, Subramanian SV (2018). Social epidemiology for the 21st century. Soc Sci Med.

[16] Hasle P, Kristensen T, Møller N, Olesen K (2007). Organisational social capital and the relations with quality of work and health—a new issue for research.

[17] Harper R (2002). The measurement of social capital in the United Kingdom. Office for National Statistics.

[18] Kawachi I, Kim D, Coutts A, Subramanian SV (2004). Commentary:reconciling the three accounts of social capital. Int J Epidemiol.

[19] Hofmeyer A, Marck PB (2008). Building social capital in healthcare organizations:thinking ecologically for safer care. Nurs Outlook.

[20] Török E, Clark AJ, Jensen JH, Lange T, Bonde JP, Bjorner JB (2018). Work-unit social capital and long-term sickness absence:a prospective cohort study of 32 053 hospital employees. Occup Environ Med.

[21] Labriola M (2008). Conceptual framework of sickness absence and return to work, focusing on both the individual and the contextual level. Work.

[22] Allesøe K, Holtermann A, Rugulies R, Aadahl M, Boyle E, Søgaard K (2017). Does influence at work modify the relation between high occupational physical activity and risk of heart disease in women?Int Arch Occup Environ Health.

[23] Devereux JJ, Vlachonikolis IG, Buckle PW (2002). Epidemiological study to investigate potential interaction between physical and psychosocial factors at work that may increase the risk of symptoms of musculoskeletal disorder of the neck and upper limb. Occup Environ Med.

[24] Clays E, Casini A, Van Herck K, De Bacquer D, Kittel F, De Backer G (2016). Do psychosocial job resources buffer the relation between physical work demands and coronary heart disease?A prospective study among men. Int Arch Occup Environ Health.

[25] Villumsen M, Holtermann A, Samani A, Madeleine P, Jørgensen MB (2016). Social support modifies association between forward bending of the trunk and low-back pain:cross-sectional field study of blue-collar workers. Scand J Work Environ Health.

[26] Kivimäki M, Head J, Ferrie JE, Shipley MJ, Vahtera J, Marmot MG (2003). Sickness absence as a global measure of health:evidence from mortality in the Whitehall II prospective cohort study. BMJ.

[27] Oksanen T, Kivimäki M, Kawachi I, Subramanian SV, Takao S, Suzuki E (2011). Workplace social capital and all-cause mortality:a prospective cohort study of 28,043 public-sector employees in Finland. Am J Public Health.

[28] Uchino BN (2006). Social support and health:a review of physiological processes potentially underlying links to disease outcomes. J Behav Med.

[29] Hvidtfeldt UA, Bjorner JB, Jensen JH, Breinegaard N, Hasle P, Bonde JP (2017). Cohort Profile:The Well-being in HospitAL Employees (WHALE) study. Int J Epidemiol.

[30] Jensen JH, Flachs EM, Skakon J, Rod NH, Bonde JP (2019). Longitudinal associations between organizational change, work-unit social capital, and employee exit from the work unit among public healthcare workers:a mediation analysis. Scand J Work Environ Health.

[31] Kiss P, De Meester M, Kristensen TS, Braeckman L (2014). Relationships of organizational social capital with the presence of “gossip and slander,”“quarrels and conflicts,”sick leave, and poor work ability in nursing homes. Int Arch Occup Environ Health.

[32] Arbejdsmiljørådet.

[33] Greenland S, Pearl J, Robins JM (1999). Causal diagrams for epidemiologic research. Epidemiology.

[34] Hansen AK, Madsen IE, Thorsen SV, Melkevik O, Bjørner JB, Andersen I (2018). Does workplace social capital protect against long-term sickness absence?Linking workplace aggregated social capital to sickness absence registry data. Scand J Public Health.

[35] de Mutsert R, Jager KJ, Zoccali C, Dekker FW (2009). The effect of joint exposures:examining the presence of interaction. Kidney Int.

[36] Vandenbroucke JP, von Elm E, Altman DG, Gøtzsche PC, Mulrow CD, Pocock SJ, STROBE Initiative (2007). Strengthening the Reporting of Observational Studies in Epidemiology (STROBE):explanation and elaboration. Epidemiology.

[37] VanderWeele Tyler J, Knol Mirjam J (2014). A Tutorial on Interaction. Epidemiologic Methods.

[38] Knol MJ, VanderWeele TJ, Groenwold RH, Klungel OH, Rovers MM, Grobbee DE (2011). Estimating measures of interaction on an additive scale for preventive exposures. Eur J Epidemiol.

[39] Gjesdal S, Ringdal PR, Haug K, Maeland JG (2004). Predictors of disability pension in long-term sickness absence:results from a population-based and prospective study in Norway 1994-1999. Eur J Public Health.

[40] Kouvonen A, Oksanen T, Vahtera J, Stafford M, Wilkinson R, Schneider J (2008). Low workplace social capital as a predictor of depression:the Finnish Public Sector Study. Am J Epidemiol.

[41] Oksanen T, Kawachi I, Jokela M, Kouvonen A, Suzuki E, Takao S (2012). Workplace social capital and risk of chronic and severe hypertension:a cohort study. J Hypertens.

[42] Oksanen T, Kouvonen A, Vahtera J, Virtanen M, Kivimäki M (2010). Prospective study of workplace social capital and depression:are vertical and horizontal components equally important?J Epidemiol Community Health.

